# Spleen and Liver Stiffness Evaluation by ARFI Imaging: A Reliable Tool for a Short-Term Monitoring of Portal Hypertension?

**DOI:** 10.1155/2022/7384144

**Published:** 2022-09-09

**Authors:** Andreas Binzberger, Mark Hänle, Matthias Pfahler, Wolfgang Kratzer, Thomas Seufferlein, Eugen Zizer

**Affiliations:** ^1^Department of Gastroenterology, Nephrology and Endocrinology (Internal medicine I), University Hospital Ulm, Germany; ^2^Department of Anesthesiology, University Hospital Ulm, Germany

## Abstract

**Background:**

Assessment of hepatic venous pressure gradient (HVPG) is the most reliable, though invasive method for evaluation of portal hypertension. Non-invasive, elastography-based techniques are well established in diagnosis, but not in monitoring of portal hypertension. The aim of our prospective study was to determine the value of acoustic radiation force impulse (ARFI) elastography technique of the liver and spleen in diagnosis and monitoring of portal hypertension.

**Methods:**

We prospectively assessed portal hypertension by HVPG and corresponding elastography of the liver and spleen in 31 patients with liver cirrhosis and an indication for primary prophylaxis by non-cardio selective beta-blockers. Investigations were performed at baseline and a follow-up visit after 6-8 weeks. To address the known large variability of values for spleen elastography, well-defined corresponding areas in the spleen were used for baseline and follow-up elastography. Sensitivity, specificity, and AUC-ROC values for both spleen and liver elastography monitoring of portal hypertension were calculated.

**Results:**

Liver but not spleen elastography significantly correlated with HVPG results and was suitable for initial evaluation of portal hypertension. However, changes in HVPG results did not show any correlation with alterations of ARFI values from baseline to follow-up visits both for liver and spleen elastography. Spleen stiffness results were not homogeneous across the whole organ differing significantly between the upper, hilar, and bottom placed investigation areas.

**Conclusions:**

In this prospective study ARFI-based assessment of liver elastography showed itself suitable for initial assessment but not for monitoring of portal hypertension. Spleen elastography was not appropriate for both, evaluation and monitoring of portal hypertension. A possible explanation for this new data that are in some contrast to previously published results is the degree of portal hypertension in our study, a comparatively short follow-up period, and well-defined investigation areas for spleen elastography in repetitive ARFI investigations. This trial is registered with NCT03315767.

## 1. Introduction

Chronic liver diseases result in structural alterations in the liver with increasing fibrosis ultimately resulting in cirrhosis. Dependent on the kind of hepatopathy fibrosis can progress fast (e.g., due to a chronic hepatitis E-virus infection in an immunosuppressed patient) or it can be a slowly progressive disease (e.g., in a patent with a nonalcoholic fatty liver disease) [1, 2]. The consequence of increasing fibrosis and alterations in the sinusoidal liver structure is portal hypertension (PH) with life-threatening complications like variceal bleeding, ascites and bacterial translocation, hepatic encephalopathy, and potentially liver cancer [3, 4]. All these events constitute decompensation of liver cirrhosis and portal hypertension with consequently increased morbidity and mortality [5, 6]: 20% of all the patients with liver cirrhosis have ascites as a first clinical manifestation of decompensated liver cirrhosis, associated with a 20% mortality within the next 12 months [7].

Hepatic venous measure gradient (HVPG) is calculated as the difference between the pressure in the portal vein and the pressure in the liver vein. Results of a HVPG investigation being >5 mmHg define portal hypertension and being >10 mmHg are associated with a clinically significant portal pressure, respectively [8]. Measurement of the hepatic venous pressure gradient (HVPG) is today the standard-of-care procedure for the assessment of portal hypertension showing an excellent correlation with the risk of decompensation of liver cirrhosis [9]. In this regard, HVPG has shown to be a very reliable method in the monitoring of non-selective beta-blocker (NSBB) therapy, and even in effectiveness assessment of a recently initiated treatment [10]. The reliable assessment of the response to the treatment of portal hypertension and an accurately timed adjustment of medication are essential for prognosis of liver cirrhosis [11]: this issue should be paid more attention because the treatment of portal hypertension by NSBB therapy can improve the portal hypertension and survival of a patient with liver cirrhosis on the one hand [12]. On the other hand, NSBB medication can worsen the prognosis of a patient with liver cirrhosis as well [13], dependent on the clinical parameters, e.g., low arterial pressure or reduced heart rate. Hence, finding a method for an accurate surveillance of patients with significant portal hypertension may help us to define a way for a successful prevention of decompensation and complications of ACLD for patients in compensated state of the liver cirrhosis. Furthermore, the performance of a HVPG intervention can be combined with a transjugular biopsy of the liver in patients with ascites or reduced platelets count who are at a significant higher risk of bleeding when liver biopsy is performed transcutaneously [14]. HVPG is an invasive procedure that itself has a periinterventional risk of complications like bleeding, infection, or thrombosis [3]. Considering that not only the performance of HVPG investigation but the interpretation of the results requires experienced hepatologists, respectively, this intervention is predominantly performed in large medical centers with expertise in advanced liver diseases. For these reasons, clinical research has been focused on identifying non-invasive markers for assessment of portal hypertension in correlation with HVPG results [15].

Evaluation of portal hypertension by assessment of blood results was performed in a considerable number of studies: a combination of albumin/ALT/INR score completed by clinical signs of advanced liver disease (spider naevi) results in excellent sensitivity for portal hypertension [16]. However, the assessment of portal hypertension by blood marker scores is frequently lacking good specifity [16, 17]. Elastography evaluation of the liver has shown a good correlation between significant liver stiffness and biopsy-assessed fibrosis/cirrhosis and showed some promising results in detecting portal hypertension. Further studies revealed a better correlation between spleen stiffness measurement (SSM) and HVPG in the diagnosis of PH [18, 19]. This led to the notion that liver elastography (liver stiffness measurement = LSM) is less suitable for a non-invasive evaluation (non-invasive test = NIT) of PH and cannot replace HVPG as also stated in the current EASL Clinical Practice Guidelines [20]. Splenic elastography became increasingly important and was identified in further studies as a robust surrogate marker for portal hypertension. However, most of the trials focused on the role of SSM and LSM in primary diagnosis of portal hypertension [21]. There are only few data on the correlation between SSM or LSM values with HVPG measurements at baseline and in response to NSBB treatment [22]. Our trial prospectively examined whether success or failure of an initiated NSBB medication can be accurately monitored by LSM- and SSM-based ARFI elastography compared with the current gold standard, HVPG.

## 2. Methods

### 2.1. Institutional Review Board

The trial protocol was approved by the local ethics committee (University of Ulm, reference number 118/16; NCT03315767). The trial conformed to the declaration of Helsinki [23] and guidelines for Good Clinical Practice in clinical trials. The participation in this trial was voluntary. All patients signed a written informed consent prior to inclusion in the study.

### 2.2. Study Patients and Design

**Figure 1:**
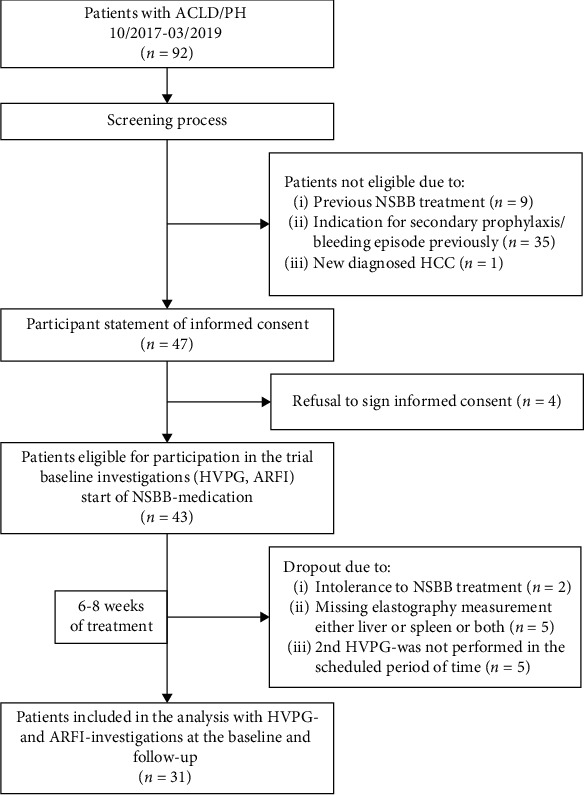
Flowchart for the study enrollment process (NSBB = non-selective beta-blocker; HCC = hepatocellular carcinoma; HVPG = hepatic venous pressure gradient; ARFI = acoustic radiation force impulse).

92 consecutive patients with clinical signs of portal hypertension and advanced chronic liver disease were screened for participation in this study in the University Hospital Ulm, Department of Gastroenterology, Nephrology, and Endocrinology (Internal medicine I) between 10/2017 and 03/2019. Considering inclusion and exclusion criteria, 47 patients with indication for primary prophylaxis by NSBB medication were offered to participate in the study with HVPG-based response monitoring scheduled. Due to refusal to sign informed consent (*n* =4), intolerance to NSBB treatment (*n* =2), or trial investigations not completed (*n* =10), 31 patients were enclosed in the final analysis with baseline and follow-up visits completed. The flowchart for enrollment process and schedule of study investigations is presented in Figure 1.

Inclusion criteria were age 18 to 80 years old, presence of varices in the upper gastrointestinal tract with indication for primary prophylaxis, no variceal bleeding in the patient history, liver cirrhosis confirmed in a biopsy or a radiologic scan, and initial HVPG >10 mmHg. Exclusion criteria were preexisting NSBB or SBB medication, an episode of a variceal bleeding or endoscopic therapy of varices previously, portal hypertension linked to another etiology but advanced chronic liver disease, decompensated liver cirrhosis, ascites or obesity making the results of liver or spleen elastography unreliable, diagnosis of hepatocellular carcinoma (HCC) or metastases in the liver, intolerance to NSBB medication due to special clinical conditions (heartbeat <60/min, atrioventricular block, obstructive pulmonary disease, systolic blood pressure results <90 mmHg), and renal insufficiency.

### 2.3. Trial Investigations

Baseline investigations comprised an assessment of portal hypertension by HVPG, evaluation of spleen and liver elastography (ARFI spleen = SSM, ARFI liver = LSM), and laboratory results, respectively. The NSBB medication was started with carvedilol at initial dose of 3,125 mg twice a day. The initial monitoring of side effects due to carvedilol and dosage adjustment was performed while patients were hospitalized and continued in the visits in our outpatient clinic. Frequency of the first clinical follow-up visits was scheduled dependent on the tolerance of the new medication and clinical conditions of the respective patient up to twice a week if necessary. In the outpatient follow-up visits, measurements of heart rate and blood pressure were regularly performed. Patients were told to perform further heart rate and blood pressure controls at home and inform the attending physician about the results for dosage adjustment. The dose for carvedilol was adjusted over the following 2 weeks until the highest tolerated medication was reached (heart rate >55/min, no sign of dizziness or hypotension with systolic blood pressure <90mmHg). The mean dosage for carvedilol used in our trial was 13.25 mg/d (6.25–25 mg/d). The follow-up investigations (HVPG and ARFI) were scheduled 6-8 weeks later.

The investigations were performed as described previously and will be briefly discussed in the following section:

- HVPG (hepatic venous pressure gradient)

The HVPG investigations were performed by experienced gastroenterologists/hepatologists (>100 previous HVPG investigations). The procedures were done according to established standards [3]. Briefly, after local anesthesia, a catheter-introducer was put in the internal jugular vein (predominantly right internal jugular vein) by Seldinger-technique. A balloon-tipped catheter (7 French) was placed in the right or middle liver vein in a fluoroscopy-guided way. Portal hypertension was assessed by measuring the transhepatic pressure gradient defined by the difference between the free and the wedged pressure in the liver vein. In every investigation, HVPG pressure was recorded three consecutive times and the mean value was calculated. HVPG values >5 mmHg defined portal hypertension per se and >10 mmHg defined clinically significant portal hypertension (CSPH), respectively. The response to NSBB medication was defined by a reduction of the HVPG value below 12 mmHg or >10% of the baseline HVPG result according to BAVENO VI recommendations for primary prophylaxis [24].

(ii) Liver and spleen elastography (ARFI technique)

All the elastography procedures were performed by physicians with specialization in ultrasound investigation and with experience in >100 prior elastography investigations (ARFI) of the liver and spleen. Ultrasound examiners were blinded regarding the results of the corresponding HVPG investigation. The evaluation of liver and spleen stiffness was performed within 24 hours of completion of the HVPG. A Siemens Acuson S3000 (Siemens Medical Solutions USA, Inc.) with a 6C1 convex transducer (1.0-6.0 MHz) was used for all ARFI examinations. The probe was placed in the 6^th^ or 7^th^ intercostal space, and it was ensured there were no major vessels or (liver) bile ducts in the B-scan in the area examined. All the recordings were performed in mid-respiratory position. The examination protocol required a measurement depth of at least 2 cm below the liver capsule or at least 5 cm from skin level. ARFI results were measured ten times in the liver and a mean value for each patient was calculated. For spleen elastography, 5 measurements were performed for the upper, the hilar, and the bottom third of the spleen, respectively, and a mean value was calculated for all the measurements in the spleen. In addition, mean values for the upper, the hilar, and the bottom third of every investigated spleen were calculated separately. For ARFI-based evaluation of the upper third of the spleen, a region of interest was mandatory set in a depth ≥5 cm; for the hilar third of the spleen, ARFI was performed in 5 cm depth and for the lower third ≤5 cm, respectively.

### 2.4. Statistical Analysis

Continuous variables were expressed as median and minimum–maximum values; categorical variables were expressed as numbers (and percentages). For the correlation between HVPG and elastography results before and after initiation of NSBB therapy, the Pearson correlation coefficient was calculated. The two-sided *t*-test was used for values with normal distribution (the Shapiro-Wilk test performed). Fisher's exact test was used for determination of possible association of categorical variables. A binary logistic regression analysis was used to identify factors significantly associated with the response in a HVPG measurement. AUC-ROC and 95% CI were calculated for changes in liver and spleen elastography (continuous variable). In all the calculations, *p*-value <0.05 was defined to be significant. All the statistical analyses were performed using R Statistical Software (version 2.14.0; R Foundation for Statistical Computing, Vienna, Austria) for Windows.

## 3. Results

### 3.1. Baseline Data

31 NSBB therapy-naive patients with advanced chronic liver disease (ACLD), a HVPG-diagnosed portal hypertension and indication for primary prophylaxis, were included in this trial. The baseline characteristics of the patients are presented in Table 1. Alcohol intake was predominantly responsible for development of liver cirrhosis (45%, *n* =14). The median Model for End-Stage Liver Disease (MELD) score at baseline was 12.0 pts (9.0–15.5 pts.). 17 patients (55%) had Child A and 14 patients (45%) had Child B cirrhosis.

### 3.2. Results of the HVPG Examinations

**Figure 2:**
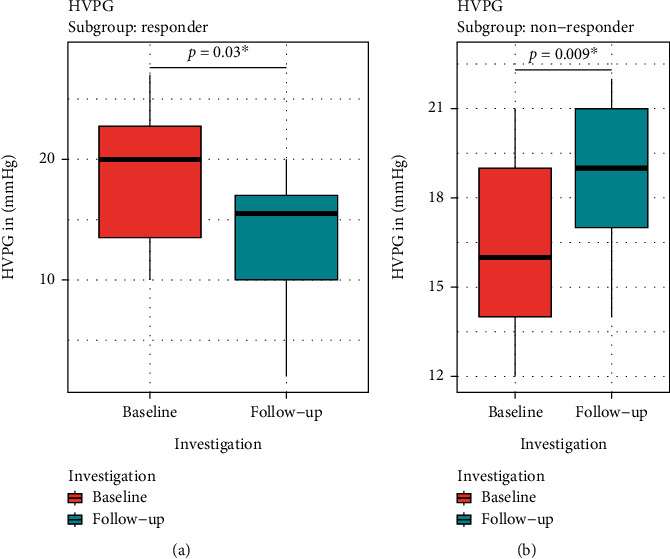
Results of the baseline and follow-up HVPG measurements in the responder and the non-responder group (a = responder, b = non-responder, red = baseline, blue = follow-up, ^∗^ = significant result) showing a significant decrease in HVPG results in the responder group (HVPG = hepatic venous pressure gradient).

Baseline HVPG examinations showed a median gradient of 18 mmHg (10–27 mmHg). In 14 (45%) patients, a treatment response could be detected in the follow-up HVPG investigation, and 17 (55%) were NSBB non-responder. In patients with response to NSBB therapy, the median HVPG value significantly dropped from 20 (10-27) mmHg at baseline to 15.5 (2–20) mmHg (-29% of baseline results; *p* =0.03; Figure 2) in the follow-up visit. In contrast, non-responder patients showed even a significant increase in HVPG values: Median HVPG was 16 (12-21) mmHg at baseline and 19 (14–22) mmHg (+16% of baseline; *p* =0.009) at the follow-up visit (Figure 2).

### 3.3. Results of ARFI Elastography of the Liver and Spleen

**Figure 3:**
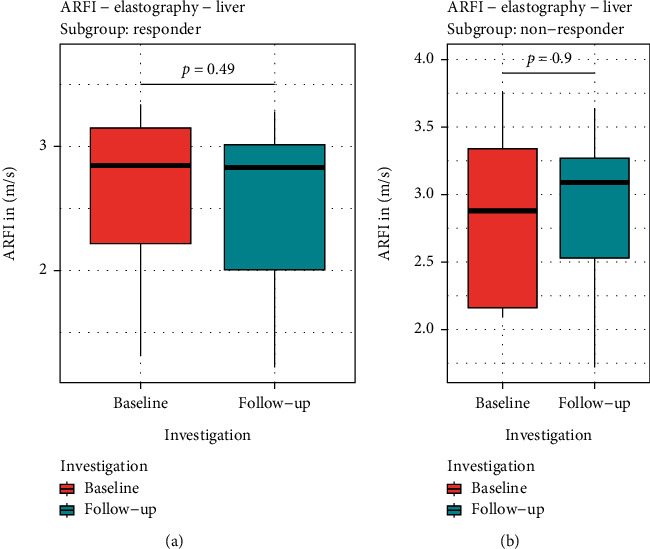
ARFI elastography results for liver stiffness (LSM) evaluation: LSM did not show any significant alterations dependent on response (a) or non-response (b) status defined by corresponding HVPG results (red = baseline, blue = follow-up) (LSM = liver stiffness measurement; HVPG = hepatic venous pressure gradient).

The baseline results for LSM showed a median value 2.88 (1.31–3.76) m/s (Table 2). In patients with HVPG responder status due to NSBB medication, a non-significant reduction of LSM results was observed (*p* =0.49; Figure 3): 2.85 (1.31–3.34) m/s (baseline) to 2.83 (1.22–3.28) m/s (follow-up). Patients without response to NSBB medication did not show any significant change in LSM values either: 2.88 (2.09–3.76) m/s (baseline) vs. 3.09 (1.72–3.64) m/s (follow-up visit, *p* =0.9; Figure 3).

The baseline results for SSM of the whole spleen showed a median value 3.00 m/s (2.27–3.59). Neither patients with response nor patients with non-response to NSBB medication showed any significant changes in spleen stiffness evaluation: 2.96 (2.27–3.34) m/s (baseline) vs. 3.02 (2.33–3.22) m/s (follow-up) in the responder group (+2% of the baseline value); 3.23 (2.38–3.59) m/s (baseline) vs. 3.03 (2.41–3.55) m/s (follow-up visit) in the non-responder group (-7% of the baseline value) (Table 2).

**Figure 4:**
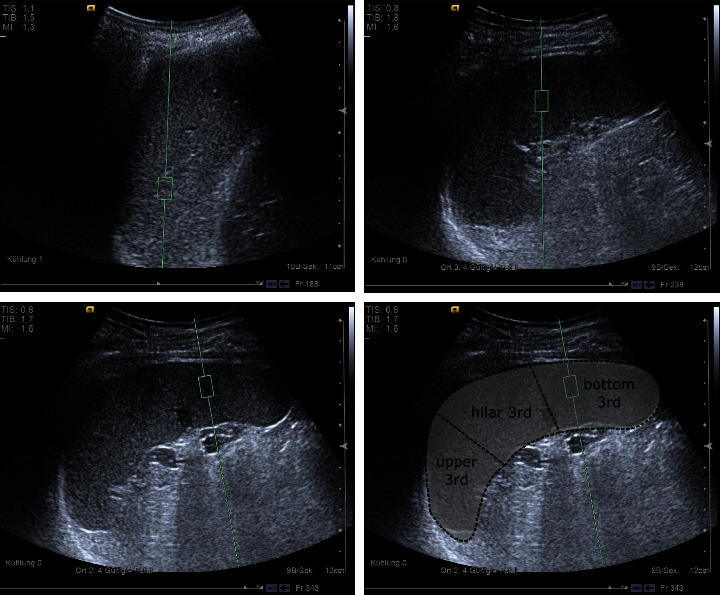
Considering significant variability of spleen stiffness results across the spleen, repetitive spleen stiffness measurements (SSM) were performed in three different sections of the spleen in the baseline and follow-up visits and respective delta results were calculated for every third of the spleen. The ARFI results were assessed in the upper, the hilar, and the bottom section (schematic image of the three spleen sections separated from each other) (ARFI = acoustic radiation force impulse imaging; SSM = spleen stiffness measurement).

**Figure 5:**
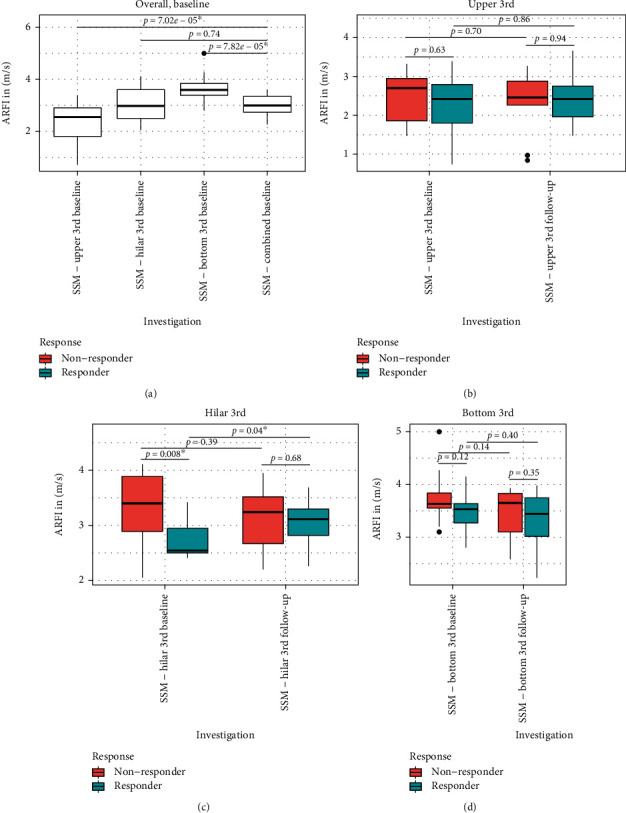
ARFI elastography results for spleen stiffness evaluation: results of SSM investigations performed on three defined spots of the spleen showed significant differences between the three positions in the spleen investigated (a). The results of spleen elastography investigations in the upper, hilar, and bottom third, respectively (red = baseline, blue = follow-up, ^∗^ = significant result) did not show a significant reduction of spleen stiffness in the response group (b/c/d) (ARFI = acoustic radiation force impulse imaging; SSM = spleen stiffness measurement).

Previously published results demonstrated a variability of stiffness results depending on the location of ARFI investigation in the spleen [25]. Therefore, we performed ARFI SSM measurements at three defined areas in the spleen (Figure 4) both at baseline and follow-up visits and compared the values obtained in these regions with the SSM values for the whole spleen: ARFI values for the upper (2.55 m/s) and bottom sections (3.59 m/s) of the spleen differed significantly from the SSM results of the whole spleen (3.00 m/s, *p* = 0.00007 and *p* = 0.00008, respectively). The ARFI results assessed in the hilar section (2.98 m/s) and the SSM results of the whole spleen (3.00 m/s) did not differ significantly (*p* = 0.74) (Figure 5 and Table 2).

### 3.4. Relationship between the Response to NSBB Therapy and the Stiffness Results of the Liver and Spleen

**Figure 6:**
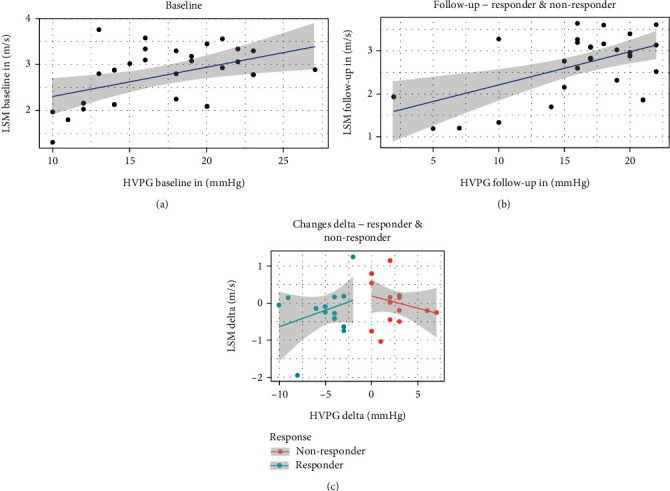
Results for liver elastography and corresponding HVPG results: LSM results showed a moderate/good correlation with corresponding HVPG results in both baseline (a) and follow-up investigations (b). *Δ*-HVPG and *Δ*-LSM values did not show any significant correlation both in response and non-response group (c) (HVPG = hepatic venous pressure gradient; SSM = spleen stiffness measurement; LSM = liver stiffness measurement).

In the responder group, there was a significant correlation (*r* = 0.82, *p* = 0.0003) at baseline visit between HVPG values and LSM results. This correlation was also detectable follow-up (*r* = 0.73, *p* = 0.0026). However, there was only a weak, non-significant correlation between the changes in HVPG and LSM values from baseline to follow-up (*Δ*-HVPG and *Δ*-LSM; *r* = 0.4, *p* = 0.15, Figure 6).

**Figure 7:**
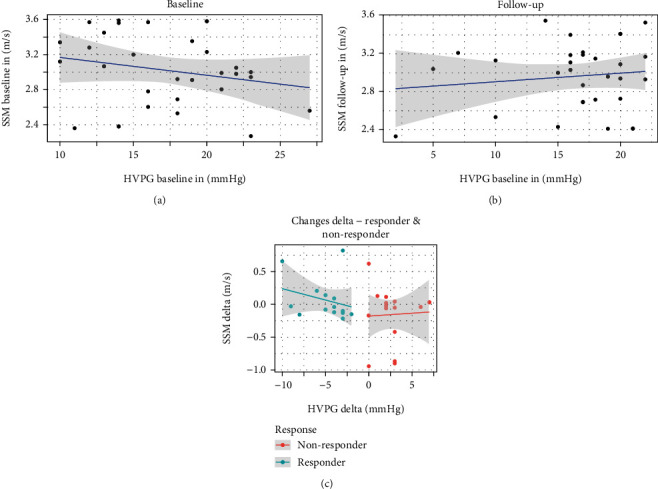
Results for spleen elastography measurement (SSM) and corresponding HVPG results: SSM results showed only a poor/no correlation with corresponding HVPG results in both baseline (a) and follow-up (b) investigations. *Δ*-HVPG and *Δ*-SSM values did not show any significant correlation both in response and non-response groups (c) (HVPG = hepatic venous pressure gradient; SSM = spleen stiffness measurement).

ARFI investigations of the whole spleen did not show any significant correlation with the HVPG results both at baseline and at the follow-up examination (*r* = -0.21, *p* =0.239 at baseline visit; *r* = 0.12, *p* =0.49 at follow-up). *Δ*-SSM and *Δ*-HVPG results between the baseline and the follow-up visits did not show any correlation either (*r* = -0.298; *p* =0.114) (Figure 7).

Considering the significantly different values in three sections of the spleen, we compared results of every section with HVPG values at baseline and follow-up visits and response status, respectively: SSM results of each of the spleen sections investigated did not show any significant, positive correlation with HVPG results or response status (Figure 6). There was also no positive correlation between changes in spleen stiffness values in all the three sections and the corresponding *Δ*-HVPG results (Table 2).

**Figure 8:**
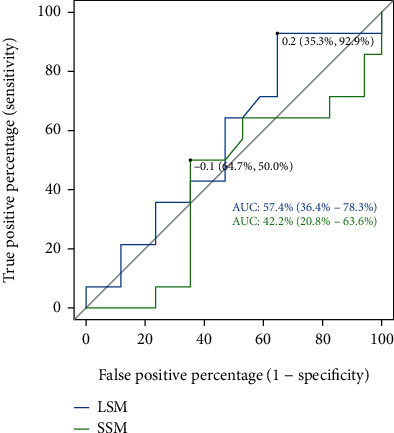
The AUC-ROC results for LSM and SSM in diagnosis of responder status in comparison to HVPG results showed a very poor ability to diagnose a successful NSBB treatment (HVPG = hepatic venous pressure gradient; LSM = liver stiffness measurement; SSM = spleen stiffness measurement).

Next, we evaluated if alterations in LSM values can help to diagnose the response to NSBB treatment compared with HVPG-based evaluation of portal hypertension at baseline and in the follow-up visit: here, the AUC-ROC result showed a very low ability of delta-LSM to identify a response to initiated NSBB medication compared to delta-HVPG (57.35%). The AUC-ROC result for delta-SSM was comparably low (42.23%) (Figure 8).

A binary logistic regression analysis of patients' data did not reveal any of the parameters being associated with HVPG-defined response. Especially, elastography values of the liver and spleen were not significantly associated with HVPG response (Table 3). Interestingly, a subgroup analysis of patients with HVPG-defined response but with negative or unchanged elastography values in the corresponding liver and spleen stiffness evaluation showed a significant association with high BMI values (>27 kg/m^2^) and obesity condition (*p* = 0.016 and *p* = 0.046, respectively).

## 4. Discussion

The results of our investigations showed that a non-invasive approach based on ARFI elastography does not accurately mirror HVPG-evaluated changes in portal hypertension in response to NSBB treatment. Liver elastography was able to detect portal hypertension and showed a strong correlation with measured HVPG values at baseline. However, in the follow-up investigation, delta-LSM was not able to allow a diagnosis of a successful response to initiated NSBB therapy compared with HVPG as gold standard. Furthermore, ARFI elastography of the spleen, performed in well-defined areas of investigation, showed only a poor performance for the diagnosis of significant portal hypertension as well as for the evaluation of the response status after NSBB treatment. In summary, stiffness assessment of both the liver and spleen by ARFI elastography was not able to reliably determine the response status to initiated NSBB therapy.

Assessment of portal hypertension has attracted growing attention in the last decades due to its increasing prognostic and therapeutic clinical impact. Several studies demonstrated a strong correlation between the incidence of liver cirrhosis related complications, like variceal bleeding, hepatocellular carcinoma, ascites decompensation, and the level of portal hypertension [26, 27]. Furthermore, invasive evaluation of portal hypertension by HVPG investigation helped us to define a response to treatment of portal hypertension with a consecutive significant decrease of cirrhosis-linked complications [24]. Due to the complex character of HVPG (invasive technique, exposure to radiation, expertise required), research has focused on evaluation of facile, non-invasive techniques for assessing and monitoring portal hypertension: elastography techniques (e.g., transient elastography (TE); acoustic radiation force impulse imaging (ARFI)) showed so far the most promising results in detecting portal hypertension and predicting consecutive complications. Liver TE has shown a good correlation with HVPG-based evaluation of clinically significant PH (CSPH; HVPG>10mmH), particularly in patients with viral hepatitis [28, 29]. In some trials, spleen TE has been demonstrated to be more reliable for the detection of significant portal hypertension compared with liver TE [30]. Recently, real-time elastography has been shown to be a considerable alternative to TE technique in the detection of portal hypertension, both by evaluation of liver and spleen stiffness [19, 31, 32]. In our investigations, despite the initially promising correlation between LSM and HVPG values, the final results did not allow any considerable correlation between *Δ*-HVPG and *Δ*-LSM with only little changes detectable, consistent with the result of other studies [33, 34]. Furthermore, the analysis of our data did not reveal any parameter we recorded (both elastography values and patients' characteristics) significantly associated with the HVPG-defined response in the follow-up investigation. Remarkably, only few data about elastography techniques have been published evaluating the detection of portal hypertension and monitoring the response after initiated treatment by NSBB. However, monitoring of changes in portal pressure after initiation of NSBB treatment and a reliable evaluation of the treatment response status are essential for patients with advanced liver disease and portal hypertension [11]: according to the NSBB response, an accurate adaptation of medication can be performed to improve survival of patients with liver cirrhosis. In consideration of this clinical benefit a few studies previously assessed changes in liver and spleen stiffness by TE [35], shear wave elastography [22], and acoustic radiation force impulse imaging (ARFI) [36]. In the results of the Korean trial by Kim et al. [36], delta-SSM (0.33 m/s) was identified being the only significant predictor of HVPG-assessed response to NSBB treatment. Interestingly, the baseline results of SSM were similar to our results in the baseline investigation (SSM 3.13 m/s vs. 3.00 m/s). But in contrast to the results of the Korean trial, our data do not support an excellent correlation between changes in spleen stiffness and HVPG values. We were not able to observe a significant decrease in spleen stiffness in NSBB responders nor we were able to demonstrate a good sensitivity and/or specificity in predicting a hemodynamic response (AUC 0.422 in our results) by spleen elastography. The difference between our study and the study of Kim et al. and the other studies investigating spleen stiffness may be explained by the way elastography evaluation of the spleen was performed: in the previous studies, delta-SSM values were not evaluated examining spleen stiffness repetitively on the same region of the spleen, but on different spots at the baseline and follow-up investigations. However, in a recently published study, a significant variation of results for spleen ARFI elastography was demonstrated depending where and how a “region of interest” is placed [25]. The results of ARFI performance can significantly vary dependent on the upper, hilar, or bottom third of the spleen investigated. Our data are in-line with this observation: three different areas in the spleen delivered significantly different results for ARFI investigation making it difficult to perform a reliable correlation with *Δ*-HVPG results. Even more crucial appears the choice of well-defined anatomic areas for spleen ARFI investigation to perform repetitive measurements. Due to this issue, we defined three distinct positions for performance of repetitive elastography investigations in the most accurate and reproducible way. Using this approach, we were not able to see a positive significant correlation between *Δ*-HVPG and *Δ*-SSM. Furthermore, SSM evaluated by ARFI technique on standardized positions was not able to deliver a reliable detection of portal hypertension. This result was also obtained in some previous studies: Procopet et al. [37] described splenic elastography by 2d shear wave elastography (SWE) not being a good choice for a reliable detection of portal hypertension. In this study, SSM assessed by 2D SWE technique was performed in only 66% of all the patients in a reliable way, consecutively associated with a lower AUC-ROC result compared with LSM for detection of clinically significant portal hypertension [37]. Further trials revealed distinct conditions, e.g., obesity and ascites, being limitations for a reliable interpretation of elastography results and diagnosis of portal hypertension and advanced liver fibrosis, respectively [38–41]. This issue should be considered for a cautious interpretation of the elastography results: in our trial, a subgroup analysis of patients with HVPG-defined response obesity and high BMI was significantly associated with unchanged or even increased LSM and SSM values in the follow-up investigation. Considering our results, a stringent definition of spots in the spleen for measurement of spleen stiffness would at least be helpful to compare data from different studies. Furthermore, distinct clinical conditions, like obesity and high BMI, should be paid more attention in the interpretation of trial results because they may be a big limitation for an elastography-based surveillance of portal hypertension.

The time for the follow-up visit is also an important topic. Chronic HVPG response was evaluated in several studies with a follow-up interval for the repeated HVPG investigations >3 months after the baseline evaluation [42]. However, the longer the interval between the baseline and the follow-up HVPG measurements, the bigger is the impact of the natural course of the hepatopathy and potential regeneration processes in the liver (e.g., due to abstinence from alcohol or successful antiviral treatment) that may also impact on HVPG values [43, 44] in addition to the direct effect of the NSBB medication. The shorter the follow-up HVPG interval, the more unbiased is the impact of the initiated medication. According to some previous trials [45], the first effect on HVPG changes can be observed as soon as 4 weeks after start of oral NSBB therapy. Here, we evaluated the response status in the second HVPG investigation approximately 4 weeks after NSBB titration was accomplished: 6-8 weeks after the baseline visit.

The basic characteristics of patients enrolled in our study do not show any significant differences compared with trials regarding treatment or prophylaxis of complications due to portal hypertension and advanced liver disease [46, 47]. In fact, our patients showed common basic characteristics in etiology for advanced liver disease (a dominant part of alcohol-linked liver damage), 70% were male participants, the majority had compensated liver cirrhosis with a low MELD-score, no/little ascites, and initiation for NSBB treatment was done for primary prophylaxis without prior bleeding episodes. In summary, the characteristics of patients enrolled in our study are in-line with real-life conditions of other medical centers.

Our study has some limitations: this single-center study was performed in a rather small number of patients with an accordingly limited number of investigators for both HVPG and ARFI evaluation. However, only few prospective studies exist for monitoring of portal hypertension by elastography techniques in correlation with HVPG results, that were performed with even a smaller number of patients compared with our trial [22]. Due to “real-life” condition patients with several etiologies for liver cirrhosis were enrolled, we were consecutively not able to perform sensible subgroup analysis for the evaluation of factors impacting the response status or spleen and liver stiffness, respectively. Finally, the level of HVPG results was significantly higher compared with other studies that were showing a good correlation between “low-level” HVPG values (<12 mmHg) and elastography techniques. This might be one of the reasons for the missing correlation between HVPG-based response status and elastography results.

## 5. Conclusions

In a prospective setting, our study demonstrates a good correlation between liver stiffness and HVPG values; hence, ARFI-based assessment of liver stiffness showed itself suitable for initial assessment of portal hypertension. However, the results of our study do not support a good correlation between spleen stiffness and HVPG values in advanced portal hypertension. Furthermore, we could not confirm a sensible role of spleen and/or liver elastography for the non-invasive evaluation of response to NSBB for a short follow-up period in patients with relevant portal hypertension. The interpretation of spleen ARFI results for evaluation of portal hypertension should be done cautiously for the spleen stiffness values are strongly dependent on the area of spleen investigated. Furthermore, high BMI and obesity seem to be limiting factors for a reliable repeated evaluation of liver and spleen stiffness for the surveillance of portal hypertension. Thus, HVPG remains the gold standard examination in this situation.

## Figures and Tables

**Table 1:** General characteristics of the patients included in the study (BMI = body mass index; MELD = model for end-stage liver disease; ASH = alcoholic steatohepatitis; AICH = autoimmune-cholestatic hepatopathy; NASH = nonalcoholic steatohepatitis; HVPG = hepatic venous pressure gradient; LSM = liver stiffness measurement; SSM = spleen stiffness measurement).

Variable	Overall *N* =31	Non-responder *N* =17	Responder *N* =14	*p*-value
Age (years)	62 (40-78)	62 (43-78)	62 (40-71)	
Sex				
Female	9 (29%)	6 (35%)	3 (21%)	
Male	22 (71%)	11 (65%)	11 (79%)	
BMI (kg/m^2^)	25.2 (16.7–33.4)	26.4 (16.7–32.2)	24.8 (21.4–33.4)	
Obesity				
Underweight/normal	14 (45%)	5 (29%)	9 (64%)	
Overweight/obesity	17 (55%)	12 (71%)	5 (36%)	
Child-Pugh score				
Child A	17 (55%)	8 (47%)	9 (64%)	
Child B	14 (45%)	9 (53%)	5 (36%)	
Liver disease etiology				
NASH	5 (16%)	4 (24%)	1 (7.1%)	
ASH	14 (45%)	7 (41%)	7 (50%)	
AICH	4 (13%)	0 (0%)	4 (29%)	
Viral	2 (6.5%)	2 (12%)	0 (0%)	
Other	6 (19%)	4 (24%)	2 (14%)	
MELD (pts.)	12 (7-19)	12 (7-19)	10 (7-18)	0.3
HVPG baseline (mmHg)	18 (10-27)	16 (12-21)	20.0 (10-27)	0.2
LSM baseline (m/s)	2.88 (1.31–3.76)	2.88 (2.09–3.76)	2.84 (1.31–3.34)	0.4
SSM overall baseline (m/s)	3.0 (2.27–3.59)	3.23 (2.38–3.59)	2.96 (2.27–3.34)	0.04^∗^

**Table 2:** HVPG, LSM, and SSM results according to initiated NSBB treatment (NSBB = non-selective beta-blocker; HVPG = hepatic venous pressure gradient; LSM = Liver stiffness measurement; SSM = spleen stiffness measurement). All the values are presented as median values (min–max).

	HVPG (mmHg)	LSM (m/s)	SSM (m/s) upper	SSM (m/s) hilar	SSM (m/s) bottom	SSM (m/s) overall
*All patients*						
Baseline	18 (10-27)	2.88 (1.31–3.76)	2.55 (0.74–3.39)	2.98 (2.05–4.11)	3.59 (2.8–5.0)	3.00 (2.27–3.59)
Follow-up	17 (2–22.0)	2.89 (1.22–3.64)	2.44 (0.84–3.66)	3.21 (2.20–3.95)	3.58 (2.23–3.98)	3.03 (2.33–3.55)
Delta (value)	-1	0.01	-0.11	0.23	-0.01	0.03
Delta (% baseline)	-5.5%	0%	-4%	7%	0%	0%
Correlation with HVPG results (*r*-value)						
Baseline		**0.46 (** **p** **=0.0089**)	0.25 (*p* =0.17)	-0.12 (*p* =0.51)	0.086 (*p* =0.64)	-0.21 (*p* =0.239)
Follow-up		**0.54 (** **p** **=0.0016**)	0.1 (*p* =0.59)	0.001 (*p* =0.97)	0.13 (*p* =0.47)	0.12 (*p* =0.49)
Delta		0.16 (*p* =0.381)	-0.25 (*p* =0.17)	**-0.38 (** **p** **=0.03)**	0.06 (*p* =0.77)	-0.298 (*p* =0.114)

*Responder*						
Baseline	20 (10-27)	2.85 (1.31–3.34)	2.42 (0.74–3.39)	2.54 (2.41–3.42)	3.53 (2.8–4.15)	2.96 (2.27–3.34)
Follow-up	15.5 (2–20)	2.83 (1.22–3.28)	2.42 (1.47–3.66)	3.11 (2.26–3.69)	3.44 (2.23–3.98)	3.02 (2.33–3.22)
Delta (value)	-4.5	-0.02	0	0.57	0.09	+0.06
Delta (% baseline)	-29%	- 0%	0%	22%	-3%	+ 2%
Correlation with HVPG results (*r*-value)						
Baseline		**0.82 (** **p** **= 0.0003**)	0.27 (*p* = 0.33)	- 0.17 (*p* = 0.53)	0.11 (*p* = 0.69)	- 0.33 (*p* = 0.25)
Follow-up		**0.73 (** **p** **= 0.0026**)	0.41 (*p* = 0.14)	0.09 (*p* = 0.75)	-0.04 (*p* = 0.88)	0.28 (*p* = 0.32)
Delta		0.40 (*p* = 0.149)	- 0.15 (*p* = 0.58)	- 0.27 (*p* = 0.34)	0.01 (*p* =0.94)	- 0.27 (*p* = 0.34)

*Non-responder*						
Baseline	16 (12-21)	2.88 (2.09–3.76)	2.7 (1.47–3.31)	3.4 (2.05–4.11)	2.7 (1.47–3.32)	3.23 (2.38–3.59)
Follow-up	19 (14–22)	3.09 (1.72–3,64)	2.46 (0.84–3.27)	3.24 (2.2–3.95)	2.46 (0.84–3.27)	3.03 (2.41–3.55)
Delta (value)	3	0.21	- 0.24	- 0,16	-0,24	- 0.2
Delta (% baseline)	16%	7%	- 9%	- 5%	9%	- 7%
Correlation with HVPG results						
Baseline		0.07 (*p* = 0.76)	- 0,18 (*p* = 0.48)	0.13 (*p* =0.59)	0.13 (*p* = 0.6)	0.06 (*p* = 0.82)
Follow-up		-0.01 (*p* =0.96)	- 0.26 (*p* = 0.31)	-0.21 (*p* = 0.31)	0.25 (*p* = 0.32)	- 0.17 (*p* = 0.50)
Delta		- 0.26 (*p* = 0.31)	**- 0.49 (** **p** **= 0.04)**	0.21 (*p* = 0.4)	0.34 (*p* =0.17)	0.04 (*p* = 0.85)

**Table 3:** Binary logistic regression analysis of elastography results of both the liver and spleen, BMI, age, and MELD score did not show any significant relationship with HVPG-defined response (BMI = body mass index; HVPG = hepatic venous pressure gradient; LSM = liver stiffness measurement; MELD = model of end-stage liver disease; SSM = spleen stiffness measurement).

Parameter	OR	95% - CI	*p*-value
LSM	0.649	(0.209 - 2.018)	0.450
SSM - upper third	1.425	(0.504 - 4.030)	0.504
SSM - hilar third	0.256	(0.059 - 1.111)	0.070
SSM - bottom third	0.132	(0.012 - 1.517)	0.104
SSM - overall	0.403	(0.070 - 2.324)	0.309
Sex	1.257	(0.245 - 6.445)	0.784
Age	0.945	(0.862 - 1.036)	0.225
MELD - score	0.939	(0.777 - 1.136)	0.518
BMI	0.976	(0.788 - 1.209)	0.825

## Data Availability

The data used to support the findings of this study (detailed patients' characteristics and detailed results of investigations) are available from the corresponding author upon request.
